# Antioxidants: The Missing Key to Improved Therapeutic Intervention in Smith-Lemli-Opitz Syndrome?

**DOI:** 10.4172/2161-1041.1000119

**Published:** 2013-11-30

**Authors:** Steven J Fliesler

**Affiliations:** VA Western New York Healthcare System; Departments of Ophthalmology and Biochemistry, State University of New York- University at Buffalo; and the SUNY Eye Institute, Buffalo, New York, USA

**Keywords:** Smith-lemli-opitz syndrome, Oxysterols, Electroretino-graphic

## Abstract

Smith-Lemli-Opitz Syndrome (SLOS) is a recessive hereditary disease caused by an enzymatic defect in the biosynthesis of cholesterol. To date, the therapeutic standard of care for this disease has been cholesterol supplementation therapy. However, the efficacy of this treatment is extremely variable and, in many if not most cases, is poor. Results of studies using animal models of SLOS have suggested that cholesterol deficiencyand/or the aberrant accumulation of the immediate precursor of cholesterol (7-dehydrocholesterol (7DHC)), per se, may not be the sole culprits in the pathobiology of this disease. Rather, cytotoxic oxysterol by-products derived specifically from 7DHC are thought to be additional, significant, causative players in the disease mechanism. Based in large measure upon such studies, a recent clinical trial, comparing the therapeutic efficacyof cholesterol supplementation alone vs. combined cholesterol-antioxidant supplementation in SLOS patients, has provided extremely encouraging results that tend to both validate the proposed role of oxysterols in the pathobiology of SLOS as well as indicate an improved treatment for this and related diseases.

Smith-Lemli-Opitz syndrome (SLOS; OMIM #270400) is an autosomal recessive human disease caused by mutations in the DHCR7 gene, which encodes the enzyme 7-dehydrocholesterol reductase (DHCR7, EC1.3.1.21), the last enzyme in the cholesterol biosynthetic pathway [1,2]. Blocking cholesterol biosynthesis at that step leads to abnormal steady-state accumulation of dehydrosterols, particularly 7-dehydrocholesterol (7DHC) and 8-dehydrocholesterol (8DHC), and reduced levels of cholesterol in all bodily tissues and fluids [3]. Disease severity ranges from in utero or early postnatal death to mild symptoms; survivors typically exhibit a range of phenotypic abnormalities (dysmorphologies), as well as moderate to severe neurological, neurosensory and cognitive defects and diminished lifespan [1,2]. To date, there is no gene therapy option for SLOS; instead, the current standard-of-care is dietary cholesterol supplementation [4]. In theory this should work: not only does it provide the missing or diminished end-product of the pathway (cholesterol), but it also should provide feed-back inhibition of the *de novo* pathway, thereby inhibiting further production and aberrant accumulation of cholesterol precursors. However, in practice, the treatment has variable, and typically minimal to modest, clinical efficacy [4]. e use of statins (e.g., simvastatin), either alone [5,6] or in combination with cholesterol supplementation [7,8] also has been evaluated, with the rationale that a statin will block the *de novo* formation and accumulation of aberrant dehydrosterols, while exogenous cholesterol would, again, provide the missing requisite end-product of the pathway. However, such therapies do not appear to provide significantly improved efficacy over cholesterol supplementation alone [4]. Clearly, something critical is missing these approaches to therapeutic intervention.

Using an animal model of SLOS generated by treating normal rats with an inhibitor (AY9944) of the same enzyme that is genetically defective in SLOS, we showed that the retina undergoes progressive degeneration and that 7DHC is the dominant sterol in all tissues, whereas 7DHC is normally barely detectable in animals and humans [9]. In addition, retinas of SLOS model rats were found to contain lipid hydroperoxides; this was dramatically exacerbated by exposing the animals to intense constant light [10,11]. Notably, treatment with an antioxidant prior to intense light exposure offered dramatic protection from the light-induced damage [10]. Also, the retina and other tissues in the SLOS rat model contained appreciable amounts of oxysterols specifically derived from 7DHC, while tissues from normal age-matched control animals do not [12,13]. Some of these oxysterols have been identified in brains from a Dhcr7-knockout mouse model of SLOS [14,15], one of which (3β,5α-dihydroxycholest-7-en-6-one (DHCEO)) has been proposed as a biomarker for the disease [14]. Notably, 7DHC is the most oxygen-labile biological molecule known, e.g., oxidizing nearly seven times faster in solution than does docosahexaenoic acid, an omega-3 polyunsaturated fatty acid (PUFA) [16]. While 7DHC itself is apparently not cytotoxic, some of its oxysterol derivatives are [17]. In addition, other studies have demonstrated the formation of oxidatively modified proteins in the retina of this SLOS animal model [18], including aldehyde adducts such as 4-hydroxynonenal (HNE) and carboxyethylpyrrole (CEP), which are by-products of the oxidative degradation of *omega-6* and *omega-3* PUFAs, respectively [19,20]. Such oxidative modifications are well-known to disrupt the normal structure and function of proteins, thereby profoundly impacting cellular and systemic physiology.

While the initial defect in SLOS arises from mutation-induced enzymatic defects in DHCR7, the resulting aberrant sterol accumulation and diminished cholesterol levels in tissues cannot fully account for the range or severity of the disease phenotype. Rather, the pathobiology mechanism likely involves additional molecular sequelae, including lipid and protein oxidation, such as the formation of 7DHC-derived cytotoxic oxysterols, other lipid hydroperoxides, and proteins covalently modified with PUFA-derived oxidation by-products [21]. In addition, while dietary cholesterol supplementation provides a partial rescue of retinal function in the SLOS rat model, there is no sparing of the retinal degeneration [22]. Hence, it is proposed that what’s missing in the current standard-of-care for SLOS is antioxidants, and that a suitable combined cholesterol plus antioxidant regimen should provide a significantly improved therapeutic intervention for treating mildly to moderately affected SLOS patients [21].

In fact, based in large measure on the results of the experimental animal studies mentioned above, a clinical trial of combined cholesterol-antioxidant therapy for SLOS is currently underway at Children’s Hospital Colorado.The initial results appear promising [23] based upon electroretinographic (ERG) testing outcomes, significant improvement in retinal function has been achieved by treating children affected with SLOS with cholesterol supplementation augmented with AquADEKs^®^, a multivitamin and mineral formulation that includes several antioxidants and micronutrients, including vitamins A, D, E, and K. AquADEKs^®^ has been used previously for treatment of cystic fibrosis patients [24,25], but has been “repurposed” for off-label use in this SLOS treatment trial.

Recent additional animal-based studies have pointed to the role of oxidative stress in the SLOS disease mechanism, including the identification of 7DHC-derived oxysterols as biomarkers for the disease, such as DHCEO (3β,5α-dihydroxycholest-7-en-6-one) [26,27]. Furthermore, tissue culture studies using fibroblasts from SLOS patients as well as studies using a genetically altered mouse model of SLOS have shown that antioxidant treatment markedly suppresses the formation of such biomarkers, and also produces a normalizing trend in gene expression patterns [24].

Due caution should be exercised, however, before either prescribing or recommending antioxidant supplementation therapy for this (or any) disease. For one, there is always the tendency for patients (or their caretakers) to self-medicate with the idea that “if one pill provides potential benefit, then several pills should give even more benefit.” Information currently is lacking with regard to which particular antioxidants, alone or in combination, may be most beneficial, what are appropriate dosages and frequencies of administration, and whether SLOS patients may have increased susceptibility to the potential toxicity of this class of compounds. What is needed is a larger randomized clinical trial to evaluate the safety and efficacy of combined cholesterol-antioxidant treatment vs. cholesterol supplementation alone, with appropriate clinical metrics. e initial trial being conducted at Children’s Hospital Colorado is a promising start in that direction. It should be noted that this approach addresses only postnatal intervention, and likely will be maximally efficacious only if treatment is initiated as early as possible and may only apply to mildly to, at worst, moderately affected individuals. Combined cholesterol-antioxidant therapy is not a “cure” for SLOS, nor a prevention; it’s merely a practical approach to minimizing some of the postnatal manifestations of the disease. A true cure awaits the development of suitable gene therapies, which are presently under investigation.
